# Measurement of low‐energy backscatter factors using GAFCHROMIC film and OSLDs

**DOI:** 10.1120/jacmp.v13i6.3832

**Published:** 2012-11-08

**Authors:** Chris J. Mart, Howard R. Elson, Michael A. S. Lamba

**Affiliations:** ^1^ The Barrett Cancer Center University of Cincinnati Cincinnati OH 45219 USA

**Keywords:** backscatter factor, GAFCROMIC film, optically stimulated luminescent dosimeters, superficial X‐rays

## Abstract

Some of the lowest voltages used in radiotherapy are termed Grenz and superficial X‐rays of ~ 20 and ~ 100 kVp, respectively. Dosimetrically, the surface doses from these beams are calculated with the use of a free in‐air air kerma measurement combined with a backscatter factor and the appropriate ratio of mass energy absorption coefficients from the measurement material to water. Alternative tools to the standard ion chamber for measuring the BSF are GAFCHROMIC EBT2 film and optically stimulated luminescent dosimeter (OSLD) crystals made from ${\text{Al}}_{2}O_{3}$. The scope of this project included making three different backscatter measurements with an Xstrahl‐D3100 X‐ray unit on the Grenz ray and superficial settings. These measurements were with OSLDs, GAFCHROMIC EBT2 film, and a PTW ionization chamber. The varied measurement methods allowed for intercomparison to determine the accuracy of the results. The ion chamber measurement was the least accurate, as expected from previous experimental findings. GAFCHROMIC EBT2 film proved to be a useful tool which gave reasonable results, and Landauer OSLDs showed good results for smaller field sizes and an increasing overresponse with larger fields. The specific backscatter factors for this machine demonstrated values about 5% higher than the universal values suggested by the AAPM and IPEMB codes of practice for the 100 kVp setting. The 20 kvp measured data from both techniques showed general agreement with those found in the *BJR* Supplement No. 10, indicating that this unit's Grenz ray spectrum is similar to those used in previous experimental work.

PACS number: 87.53.Bn

## I. INTRODUCTION

The depth of penetration of X‐rays dictates their usefulness for certain applications; therefore megavoltage X‐ray beams are useful for reaching deep seated targets, whereas kilovoltage X‐rays are more appropriate for shallow targets. Some of the lowest voltages used in radiotherapy are termed Grenz and superficial X‐rays with peak voltages of ~ 20 kVp and ~ 100 kVp, respectively. Therapeutically, these beam qualities are most often used for superficial cancers and keloids with a target depth of only 0.3–1 mm below the outer surface of the skin; therefore, the dose to a target from the beam is typically quantified as a surface dose. Dosimetrically, this surface dose is calculated with the use of a free in‐air air kerma measurement combined with a backscatter factor and the appropriate ratio of mass energy absorption coefficients from the measurement material (air) to water:

(1) $$D_{w,z=0}=MN_{k}B_{w}\left[{\left(\frac{{\bar{\mu }}_{en}}{\rho }\right)}_{w/air}\right]$$

While the mass energy absorption coefficients are fairly well documented, the backscatter factors are a bit more ambiguous. A backscatter factor is defined as, “… a ratio of the kerma rate to water at the surface of the water phantom to the kerma rate to water at the same point in space in the absence of the phantom.”^(1)^

(2) $$BSF=\frac{D_{sur,fs}}{D_{air,fs}}$$

The importance of the BSF in determining the absorbed dose at the surface of a patient is clear, but achieving the accurate measurement of this factor is not simple.

The BSF measurement is confounded by several factors, depending on the type of detector that is used. For ionization chambers there are competing effects: the displaced material in the chamber volume results in a lack of scatter being created within the chamber, and also more scatter occurring posterior to the chamber due to lack of attenuation.^(2)^ There is also the concern with these chambers that extra scatter is being produced in the wall due to the physical size of the apparatus. Some of the smallest ionization chambers have a collecting volume of $0.3\;{\text{cm}}^{3}$, which results in an ambiguity of the measurement location. This is an important parameter, as the kerma changes rapidly with depth at these energies, and the measurement is assumed to be at the surface. Another detector type is the lithium fluoride thermoluminescent dosimeter (TLD) crystal. This detector can produce incorrect results due to the varied response of the TLD to different incident energies of radiation. This energy dependence is due to the effective atomic number of the LiF: approximately 8.14 vs. 7.42 for tissue. This comes into play as the primary beam and the backscattered beam have slightly different energy spectra. There is an advantage to crystal dosimeters as they have a volume of $0.01\;{\text{cm}}^{3}$, which reduces the ambiguity of the measurement location. Other alternatives are GAFCHROMIC EBT2 film (International Specialty Products, Wayne, NJ) and optically stimulated luminescent dosimeter (OSLD) crystals made from ${\text{Al}}_{2}O_{3}$. OSLDs offer the same small volume advantage as TLDs, but have greater energy dependence, with an effective atomic number of 10.65, which is on the order of <10%.^(3)^ They have a further advantage in their re‐readability, which can reduce the measurement uncertainty. EBT film exhibits less accuracy, but an even smaller volume, a lack of energy dependence and rereadability for a possibly ideal solution to making this measurement.

The measurement device is not the only confounding factor when determining the correct backscatter factor for a superficial beam. The BSF is greatly dependent on the spectral properties of the specific beam in use. An example of this dependence is shown by Johns et al.,^(4)^ who found that a lightly filtered high kVp beam has a lower BSF than a heavily filtered low kVp beam with a similar half‐value layer. In a multi‐institution survey conducted by Ma et al.,^(5)^ a 100 kVp beam was found to have many different HVLs, illustrating the fact that beam spectra vary widely from machine to machine. The spectrum of a superficial unit's output can be dependent on a number of factors such as kVp, added filtration, anode angle, inherent filtration, tube current, and field size. Thus, not only is obtaining the correct BSF difficult by measurement, but it is also difficult to utilize a published table of values, such as that by the IPEMB, to determine a value for the user's machine since the beam spectra may not be similar enough.^(6)^ This concept shows the importance of correctly characterizing the beam on which BSF measurements are made so that these values may be objectively compared to another set of data.

One of the earliest backscatter factor measurements was made by Johns et al. in 1954.^(4)^ He used a custom setup built on site that included a $0.04\;{\text{cm}}^{3}$ thimble‐type ionization chamber with carbon‐coated aluminum electrode adjusted for less than 5% energy response. His results were used to update an earlier work done by Braestrup in 1944 and were published in the *British Journal of Radiology*.^(7)^ The next advance in BSF data came with the advent of Monte Carlo simulations. Grosswendt^(8)^ published Monte Carlo data which was significantly different from the BSFs in the *BJR*. Later, Klevenhagen^(1)^ conducted experimental work using a 3 mm thick plane‐parallel ionization chamber in order to verify the Monte Carlo work of Grosswendt. Klevenhagen's work, along with the updated Monte Carlo simulations of Grosswendt and Knight, verified the new data, and an amalgamation of these three results was published in IPEMB as the new standard.^9^, ^10^ TLDs were used by Harrison to find BSF data that was not statistically different from the Monte Carlo data of Grosswendt.^2^, ^8^ Of note is the wide statistical deviation involved with using TLDs for exposure measurement as they can only be read once and are not extremely consistent. More recently, GAFCHROMIC film was tested by Kim et al.^(11)^ to determine its effectiveness in measuring BSFs. This group again found data that was not statistically different from the published data, but it was determined that EBT film could be a reliable method for measuring BSFs. Further, Kim et al. had trouble comparing their data to earlier published data, due to the different energy spectra of the units used. This serves to emphasize the point that measuring machine‐specific data is in order if the user's unit has characteristics significantly different from those used for the published data, which is likely the case for all modern superficial units.

## II. MATERIALS AND METHODS

Different backscatter measurements were made with the Xstrahl Gulmay D3100 X‐ray unit (Chamberley, Surrey, UK) on the Grenz ray and superficial settings. These measurements were with OSLDs and GAFCHROMIC EBT 2 film. The varied measurement methods allowed for intercomparison to determine the accuracy of the results. Further, the beams were extensively characterized with kVp, HVL, flatness and symmetry, anode angle/material, and filtration parameters identified. This enabled comparison of measured BSFs to published values accounting for spectral differences.

The half‐value thicknesses (HVT) were measured using a 4 cm cone and two different protocols that are specified in the AAPM Report 67 and the IPEMB report.^5^, ^6^ The TG‐61 method involves narrow beam geometry with the aluminum filter and lead collimators placed half‐way between the source and detector, along with a second monitor chamber above the collimation. The IPEMB method was similar, with the absence of collimation, resulting in broad beam geometry. Film was used to verify that both the detector and aluminum filter were aligned in the center of the field for both methods. This measurement was made with both methods for the 100 kVp quality X‐ray beam; however, as these two reports do not deal with very low‐energy X‐rays of 20 kVp, the HVT of this beam was measured using only the narrow beam geometry method for comparison with the older *BJR* data.

Beam profiles were measured using a full sheet of EBT film placed directly beneath a 10 cm cone for each of the 20 and 100 kVp settings. These films were scanned (Vidar Dosimetry Pro, VIDAR Systems/Contex Group, Stockholm, Sweden) and analyzed with film dosimetry software (RIT, Radiological Imaging Technology, Colorado Springs, CO), using profiles to check for any variation in the output across the field.

The BSF measurements with EBT film closely followed the procedure used by Kim et al.^(11)^ The film was cut into small ~ $1\;{\text{cm}}^{2}$ pieces and suspended with thin plastic wrap on the end of a cone for irradiation in the presence and absence of a liquid water tank (25×25×25 cm). When using the water phantom, the cone was placed just at the water's surface, so the film lay on top of the plastic wrap on the water's surface. When measuring the in‐air data, the film was on the inside of the plastic wrap within 1 mm of the cone's end and the cone was pointed toward the floor at a distance of 2 m. This procedure was repeated for six in‐air film measurements and six in‐water film measurements for each of the cones (2, 4, 6, 8, 10, and 15 cm) and beam energies.

**Figure 1:**
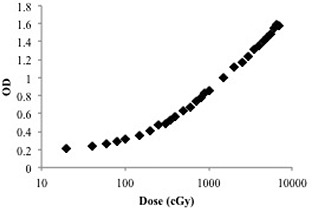
6 MV EBT2 film calibration showing film OD plotted vs. dose.

The film was also calibrated at 6 MV so the film's response could be correlated to dose. The film was irradiated at depth of maximum dose in a solid water phantom from a 6 MV therapeutic X‐ray beam to doses from 20 cGy to 7000 cGy. Though the manufacturer suggested range of doses for this film is up to 10 Gy, a much higher dose was needed to fill out the calibration curve up to saturation levels. Instead of scanning these films, their optical densities (OD) were read with a densitometer (model TBX, Tobias Associates, Inc., Warminster, PA) three times and plotted vs. dose to generate a calibration curve (see Fig. 1). The OD of the film was read three times for each of the in‐air and in‐water measurements also, to verify consistency of the results; however, it is expected that the film's response will only be precise to within ±2%.^(12)^ The average ODs of the six in‐air measurements and the average of the six in‐water measurements for each cone were plugged into the calibration equation to obtain the doses delivered to the film for each. These doses were then divided to obtain the average BSFs for each cone. The standard error for the BSFs was determined by propagating the uncertainty in the OD measurement of the film through the OD to cGy calculation and the BSF calculation for each cone size (see Tables 1and 2).

**Table 1:** 100kVp backscatter factor results. The BSF data from both film and OSLD measurements are listed along with published data from the IPEMB corresponding to the appropriate cone sizes, FSDs, and HVTs. The first row of p‐values is calculated between each of the film and OSLD data and the IPEMB values, while the second row of p‐values is calculated between the film and OSLD values.

*Cone*		*Film*	*OSLD*	*IPEMB*
2 cm		1.18	1.16	1.12
	0	0.08	0.01	
	p	0.211	0.000	
	p	0.601		
4 cm		1.21	1.21	1.20
	0	0.07	0.02	
	p	0.802	0.467	
	p	0.992		
6 cm		1.29	1.32	1.25
	0	0.07	0.01	
	p	0.262	0.000	
	p	0.291		
8 cm		1.36	1.35	1.29
	0	0.10	0.02	
	p	0.139	0.002	
	p	0.801		
10 cm		1.39	1.51	1.32
	0	0.07	0.04	
	p	0.090	0.000	
	p	0.006		
15 cm		1.28	1.48	1.37
	0	0.05	0.03	
	p	0.007	0.000	
	p	0.000		

**Table 2:** 20kVp backscatter factor results. The BSF data from both film and OSLD measurements are listed along with published data from BJR Supplement No. 10 corresponding to the appropriate HVTs. The first row of p‐values is calculated between each of the film and OSLD data and the BJR values while the second row of p‐values is calculated between the film and OSLD values.

*Cone*		*Film*	*OSLD*	*BJR*
2cm		1.10	0.97	1.01
	0	0.04	0.04	
	p	0.034	0.224	
	p	0.032		
4cm		1.12	0.99	1.02
	0	0.03	0.05	
	p	0.010	0.434	
	p	0.032		
6cm		1.00	1.01	1.03
	0	0.02	0.05	
	p	0.053	0.570	
	p	0.593		
8cm		1.07	0.96	1.04
	0	0.06	0.05	
	p	0.545	0.075	
	p	0.098		
10cm		1.02	1.07	1.05
	0	0.05	0.11	
	p	0.379	0.834	
	p	0.539		
15cm		1.03	0.86	1.06
	0	0.04	0.02	
	p	0.418	0.000	
	p	0.023		

For the second set of BSF measurements, NanoDot OSLDs (Landauer Inc., Glenwood, IL) were used. These crystals were suspended by thin plastic wrap in the same manner that the film was — with the cone pointing through air toward the floor at a distance of 2 m for an in‐air measurement and with the cone at the surface of the water for the in‐water measurement. While OSLDs don't have an exactly consistent response to radiation, their uncertainty can be reduced with multiple readings of the crystal after a single exposure. An analysis of the signal depletion due to multiple readings was performed by Jursinic^(3)^ and found to be on the order of $.9993^{n}$. This is only 0.3% over five readings, well within the uncertainty of a single reading; so obtaining multiple counts still provides an advantage. The OSLDs have a linear response of counts to dose up to roughly 20 Gy negating the need for a calibration curve.^(3)^ The BSF measurements consisted of six in‐air and six in‐water measurements, each read five times to give an average number of counts for the air and phantom data points. The BSFs were obtained by dividing the average counts for each dosimeter and averaging the results for each cone size. The standard error for the OSLD back scatter factors was calculated by propagating the uncertainty of the OSLD counts through the BSF calculation for each dosimeter and the BSF group average for the cone (see Tables 1and 2).

## III. RESULTS & DISCUSSION

### A. Film Calibration

The EBT film calibration was accomplished with a wide range of doses from 20 cGy to 7000 cGy using graduated intervals to match the exponential nature of the film darkening. The calibration curve shown in Fig. 1 was used to generate a 4th order polynomial (see Eq. (3)), which relates film OD to dose delivered. This function was found to best fit the data points with an $R^{2}$ of 0.998.

(3) $$D=113.1(OD^{4})+2675.6(OD^{3})-3123.1(OD^{2})+222.51(OD)-361.17$$

The GAFCHROMIC film calibration was performed at 6 MV and applied at the lower kilovoltage energies of the superficial machine. This calibration is believed to be applicable to the lower energies due to the low‐energy dependence of GAFCHROMIC film and the relative nature of the backscatter factor measurement.^(12)^ The curve (Fig. 1) shows a distinct linear region ranging from doses of 500 cGy to upwards of 7000 cGy; for this reason and because lower doses yielded optical density values too close to base values, exposures resulting in approximately 1000 cGy were chosen for both the in‐air and in‐water film measurements. This dose region had extremely good fit to the polynomial function, thereby allowing accurate determination of the BSF. The energy dependence of ≤10% from MV down to kilovoltage energies likely overestimates how much variation would be seen between the primary and backscattered X‐rays involved in this measurement. Additionally, absolute dose values were irrelevant, so long as the film responded similarly to both the in‐air and in‐water measurements.

### B. Half‐value thickness

The next step was to take measurements of the half‐value thickness, in order to make comparisons between measured data and published data. As discussed previously, a single parameter such as HVT is not sufficient to define a beam's spectrum; however, published data are currently categorized solely in this manner. The measurements included both AAPM and IPEMB methodologies of narrow and broad beam geometry. These techniques yielded the expected variations with the narrow beam technique producing a slightly lower HVT due to the exclusion of excess scatter being detected. As both published reports contain the same experimental and Monte Carlo data, they are categorized in the same manner. It is surprising then, that they each call for different methodologies when making the HVT measurement — methodologies which are expected to yield different results. However, as the published data only includes a few sparse HVT categories, the small difference between the methods doesn't create a large problem. For this work, the results of the two methods were averaged, see Table 3, and this value, along with field size and focal skin distance (FSD), used to draw data from the published tables.

**Table 3:** Measured half‐value thicknesses for both beam energies.

*Energy (kVp)*	*HVT (mmAl)*
100	3.97
20	.0816

### C. 100 kVp backscatter factors

The 100 kVp data has been gathered in Table 1, showing the BSF values calculated, as well as the most accurate published data available based on the field size and HVT measured. Statistical probability values have also been calculated using a two‐sided Student's t‐distribution between the two measured values and between the measured values and the published value for each cone size. The standard error for each factor is reported as well. The GAFCHROMIC film BSFs are nearly uniformly higher than the published data. The two anomalies are the 4 and 15 cm cone sizes. The 4 cm cone BSF is nearly the same as the OSLD and published data, while the 15 cm cone is statistically lower than both of the other values. The 2, 6, 8, and 10 cm cones are all approximately 5% higher than the published data, but not statistically so with p‐values greater than 0.05. The OSLD BSFs follow a similar trend when compared to the IPEMB data, with the exception of the 15 cm cone size, which shows a higher BSF instead of lower. The 4 cm cone is, as mentioned, no different than the published value, while the 2, 6, and 8 cm cones are 5% higher than the IPEMB BSFs, and the 10 and 15 cm cones are an average of 11% higher. All p‐values comparing the OLSD BSFs to the IPEMB values are less than 0.01, except for the agreeing 4 cm BSF. When comparing the film and OSLDs, the data are split, with two values being statistically different and four not different. Putting all of this together, it seems that the OSLD and film data agree that the BSF for this machine is generally higher than the published data would suggest for the smaller field sizes. For the larger cones of 10 and 15 cm, the OSLD measurement becomes increasingly higher than the GAFCHROMIC film one. Since EBT film has been shown to be capable of making an accurate BSF measurement using this method, the film value is taken as being more likely correct. The OSLDs are expected to exhibit an increased response to lower energy X‐rays from the photoelectric effect, and given that there is more backscatter (lower energy X‐rays) in the larger field sizes, the higher BSF resulting from this tool compared to film at the largest cone sizes is explained. To address the general trend of BSFs being higher than the published data, the idea of a machine‐specific BSF comes into play. The 100 kVp beam is heavily filtered with 5 mm of aluminum in addition to the thin 0.9 mm beryllium tube window. This produces a harder beam than those utilized in other machines with less filtration and higher potential, despite having similar half value thicknesses. This machine's harder beam is capable of more penetration, and harder beams in general are found to have higher backscatter factors than softer beams with similar HVTs.^(4)^

### D. 20 kVp backscatter factors

The results for the 20 kVp BSFs are shown in Table 2, along with the closest published values based on the measured HVT. Standard errors and p‐values are shown in this table that have been calculated in the same manner as for the 100 kVp data, indicating which values are statistically significant from each other. The set of comparison data from the literature comes from an older publication for this setting, as the newer data do not have values for such low HVTs. Also, it is of note that this supplement gives the 0.07mmAl HVT BSF as a simple range of 1.00–1.07 for the varying field size and focal skin distance combinations. Therefore, it is not explicitly specified that the BSF increases uniformly with field size within this given range, but it seems a reasonable assumption and has been made for this work's purposes. The 20 kVp setting uses no additional filtration beyond the beryllium window on the tube resulting in an extremely soft spectrum. The film results show some interesting trends, with the 2 and 4 cm cone backscatter factors about 9% higher than the expected values from the *BJR* report, while the rest of the cone sizes do not show any difference at the 0.05 significance level. Further, the BSFs measured with OSLDs show agreement with *BJR* data for all of the field sizes except for 15 cm. The general agreement of both sets of data with the BJR values indicate that for this setting, the universal data is sufficient and there are no disrupting factors which would render machine‐specific BSFs necessary. However, as there is general, but not universal, agreement between the measured data, the methodology is drawn into question to explain the outliers. The same technique was used for both film and OSLD measurements, but small positional differences were possible. The film or OSLD was only visually aligned to the center of the cone and the plastic wrap was applied tightly to ensure uniformity; however, the wrap becomes loose over time and with exposure to water, possibly resulting in small differences in distance between the source and the measurement device. The beam profiles that were measured with GAFCHROMIC film showed good symmetry and flatness across the entire field, indicating that lateral positional errors would not have a large effect on the measurement. The difference in distance could have a significant impact though, especially at such a low tube potential. It is likely therefore that the outlying measurements are indicators of small deviations in position, resulting in erratic BSF values.

## IV. CONCLUSIONS

Backscatter factors were measured for an Xstrahl Gulmay D3100 superficial machine on the 20 kVp and 100 kVp settings for six cone sizes using different techniques. GAFCHROMIC EBT2 film proved to be a useful tool which gave reasonable results and Landauer OSLDs showed good results for smaller field sizes and an increasing overresponse with larger fields. The specific backscatter factors for this machine proved to be about 5% higher than the universal values suggested by the AAPM and IPEMB codes of practice for the 100 kVp setting. The 20 kvp measured data from both techniques showed general agreement with those found in the *BJR Supplement*, indicating that this unit's Grenz ray spectrum is similar to those used in previous experimental work.
